# Voices of Caregivers: Key Demands Towards AI-driven Home Monitoring in Community-based Dementia Care

**DOI:** 10.1093/geroni/igab046.2495

**Published:** 2021-12-17

**Authors:** Christian Wrede, Annemarie Braakman-Jansen, Lisette van Gemert-Pijnen

**Affiliations:** 1 University of Twente, Dept. of Psychology, Health & Technology, Enschede, Overijssel, Netherlands; 2 University of Twente, Enschede, Overijssel, Netherlands

## Abstract

While most people with dementia prefer to live at home for as long as possible, this also puts more pressure on both their informal and formal care network. To provide support in home-based dementia care, there is growing interest in technology that allows caregivers to remotely monitor health and safety of people with dementia. Novel generations of these technologies are using non-wearable, pervasive sensors coupled with algorithms to continuously collect and model meaningful in-home information. However, while these self-learning monitoring systems develop rapidly, their target users’ views and demands are still insufficiently mapped out. To identify possible barriers to acceptance and ways to overcome these, we conducted a scenario-based study, including semi-structured interviews with informal caregivers (n=19) and focus groups with home care professionals (n=16) of community-dwelling people with dementia. Inductive qualitative content analysis revealed that both groups of caregivers were concerned about the informational privacy of their care recipient with dementia, information overload, and ethical issues related to dehumanizing care. Identified demands mainly centered around how to overcome these barriers. We identified several demands related to specific functionalities, user experience factors, services surrounding the technology, and integration into the existing work context. Most notably, caregivers highlighted the importance of introducing AI-driven in-home monitoring technologies in a way it prevents them from feeling undervalued. In conclusion, our findings can help to inform the development of more acceptable and unobtrusive in-home monitoring technologies to support home-based dementia care.

